# Barriers and Facilitators to the Implementation of Personalised Medicine across Europe

**DOI:** 10.3390/jpm13020203

**Published:** 2023-01-23

**Authors:** Dorota Stefanicka-Wojtas, Donata Kurpas

**Affiliations:** 1 Clinical Trial Department, Wroclaw Medical University, 50-556 Wroclaw, Poland; 2 Family Medicine Department, Wroclaw Medical University, 51-141 Wroclaw, Poland

**Keywords:** Personalised Medicine, interregional cooperation, barriers, facilitators, healthcare systems

## Abstract

(1) Background: Personalised medicine (PM) is an innovative way to produce better patient outcomes by using an individualised or stratified approach to disease and treatment rather than a collective approach to treating patients. PM is a major challenge for all European healthcare systems. This article aims to identify the needs of citizens in terms of PM adaptation, as well as to provide insights into the barriers and facilitators categorised in relation to key stakeholders of their implementation. (2) Methods: This article presents data obtained from the survey “Barriers and facilitators of Personalised Medicine implementation—qualitative study under Regions4PerMed (H2020) project”. Semi-structured questions were included in the above-mentioned survey. The questions included both structured and unstructured segments in an online questionnaire (Google Forms). Data were compiled into a data base. The results of the research were presented in the study. The number of people who participated in the survey can be considered an insufficient sample size for statistical measurement. In order to avoid collecting unreliable data, the questionnaires were sent to various stakeholders of the Regions4PerMed project, which includes members of the Advisory Board of the Regions4PerMed Project, but also speakers of conferences and workshops, and participants in these events. The professional profiles of the respondents are also diverse. (3) Results: The insights on what would help in the adaptation of Personal Medicine to citizen needs have been categorised into 7 areas of need: education; finances; dissemination; data protection/IT/data sharing; system changes/governmental level; cooperation/collaboration; public/citizens. Barriers and facilitators have been categorised into ten key stakeholders of the implementation barriers: government and government agencies; medical doctors/practitioners; healthcare system; healthcare providers; patients and patient organisations; medical sector, scientific community, researchers, stakeholders; industry; technology developers; financial institutions; media. (4) Conclusions: Barriers to the implementation of Personalised Medicine are observed across Europe. The barriers and facilitators mentioned in the article need to be effectively managed in healthcare systems across Europe. There is an urgent need to remove as many barriers as possible and create as many facilitators as possible to implement personalized medicine in the European system.

## 1. Introduction

Personalised Medicine (PM), as highlighted by Fournier et al., (2021), for example, is becoming an issue in health policy and in the media. Importantly, and highlighted by the authors, there is no consensus in the scientific literature on the definition of PM. The same term is defined emphasizing both patient-centred and biomedical aspects [1]. PM is further complicated by the synonymous use of terms such as precision, personalised, stratified and targeted medicine [2]. The term PM and what it involves, as pointed out by Botham et al., (2021), is still largely unfamiliar to the public [2], and is relatively new in medical society [3]. The Horizon 2020 Advisory Group of the European Commission defines PM as “a medical model using characterization [sic] of individuals’ phenotypes and genotypes (e.g., molecular profiling, medical imaging, lifestyle data) for tailoring the right therapeutic strategy for the right person at the right time, and/or to determine the predisposition to disease and/or to deliver timely and targeted prevention”.

Although personalised medicine is becoming the new paradigm for the management of certain diseases, the economics of personalised medicine has only focused on assessing the efficiency of specific treatments, and lacks a theoretical framework analysing the interactions between pharmaceutical companies and healthcare systems, leading to the implementation of personalised treatments [4].

PM is rooted in the belief that, since individuals possess nuanced and unique characteristics at the molecular, physiological, environmental exposure and behavioural levels, they may need interventions for diseases they suffer from that are tailored to these nuanced and unique characteristics [5].

PM is strongly developing because the influence of individual characteristics on disease progression and the efficacy of medication is becoming more evident. Some people have, due to their genetic makeup, a higher risk of severe side effects when using specific medication. Others are more sensitive to the medication and need a different dose than generally recommended. In addition, the genetic characteristics of tumours in cancers may differ from patient to patient, which creates opportunities to fine-tune the therapy based on tumour characteristics [3].

The application of PM requires having instruments (tests) to stratify patients, as well as personalised treatments [6].

The implementation of PM in clinical practice requires smart planning and a structured approach to ensure quality and long-term sustainability [7]. The integration of PM into mainstream healthcare will only be successful, as noted by Holde at al. (2019), if the public understands and supports this change [8].

Leaders of countries and major healthcare organisations are pushing for rapid translation of these discoveries into benefits for patients. However, shifting to “an innovative approach that takes into account individual differences in people’s genes, environments, and lifestyles” from the existing “one-size-fits-all” approach to healthcare requires a better understanding of this new approach by the public and other stakeholders [1].

## 2. The Aim of the Study

The aim of this study was to investigate and identify the interventions that would remove the barriers to the implementation of innovative interventions and to identify the best practices implemented in the European countries which support the implementation of innovative interventions in the field of PM.

## 3. Materials and Methods

### 3.1. Ethics Approval

This study was approved by the Bioethics Committee of the Wroclaw Medical University under number KB0450/2020. 

### 3.2. Study Design

To address the subject of the study, a semi-structured questionnaire “Barriers and facilitators of Personalised Medicine implementation—qualitative study under Regions4PerMed (H2020) project” was developed. The entire survey has been added as the Supplementary Materials File S1.

The survey included demographic questions regarding age, gender and nationality, which allowed us to better understand the background of the respondents. The survey also included two questions related to individual experiences with barriers and facilitators of PM implementation, and five questions about the implementation of PM itself.

### 3.3. Study Design

This study analyses data from the online semi-structured survey “Barriers and facilitators of Personalised Medicine implementation—qualitative study under Regions4PerMed (H2020) project”. 

For this purpose, the above-mentioned survey was designed. This semi-structured questionnaire includes items with some pre-categorised response options and additional open-ended options. It includes general information on gender and nationality, two questions on individual experiences with barriers and facilitators of PM implementation, and five questions about the implementation of PM itself. The survey responses were coded in the following format—a country symbol and a number indicating the order in which the surveys were delivered.

### 3.4. Questionnaire Development and Data Collection

#### Participants

Stakeholders of the Regions4PerMed (Interregional Coordination for a Fast and Deep Uptake of Personalised Health) project, which includes members of the Advisory Board of the Regions4PerMed Project, lecturers at conferences and workshops and participants in these events, were asked to complete the semi-structured survey.

Data were collected from 85 respondents. Interviewees came from 20 countries, including Ukraine (UA), Italy (IT), Germany (DE), Spain (ES), Poland (PL), Denmark (DK), Belgium (BE), Great Britain (GB), Latvia (LV), Canada (CA), Estonia (EE), Turkey (TR), Romania (RO), Europe (EU), France (FR), Lithuania (LT), Sweden (SE), Greece (GR), Netherlands (NL), Portugal (PT) and Kazakhstan (KZ). The age of respondents ranged between 24–74.

The participants include researchers (scientists), entrepreneurs, scientific officers, policy officers, policy advisors, project managers, physicians, civil servants, lawyers, public health experts, health care managers, biostatisticians, health care consultants, etc.

### 3.5. Variables

#### 3.5.1. Quantitative Variables

The survey included demographic information on quantitative variables, such as age, gender and nationality.

#### 3.5.2. Qualitative Variables

The survey collected responses to questions on individual experiences related to the barriers and facilitators to implementing PM, and responses to five questions on the implementation of this concept. 

In this paper, only the results that are relevant to the aim of the study are presented.

### 3.6. Data Sources

The presented research analyses is sourced from the collected data obtained from online semi–structured surveys (survey “Barriers and facilitators of personalised medicine implementation—qualitative study under Regions4PerMed (H2020) project”, author of the survey—Dorota Stefanicka—Wojtas).

### 3.7. Study Size

Between July 2020 and November 2022, 85 surveys were conducted. Semi-structured questions were included in the above-mentioned survey. The questions included both structured and unstructured segments in an online questionnaire (Google Forms). The data were compiled into a database. The results of the research were presented in the study.

## 4. Results

This section is divided by subheadings. It should provide a concise and precise description of the experimental results and their interpretation, as well as the experimental conclusions that can be drawn from the study.

### 4.1. Participants and Descriptive Data—Survey

The results from the semi-structured survey “Barriers and facilitators of Personalised Medicine implementation—qualitative study under Regions4PerMed (H2020) project” are presented below. 

**Figure 1:**
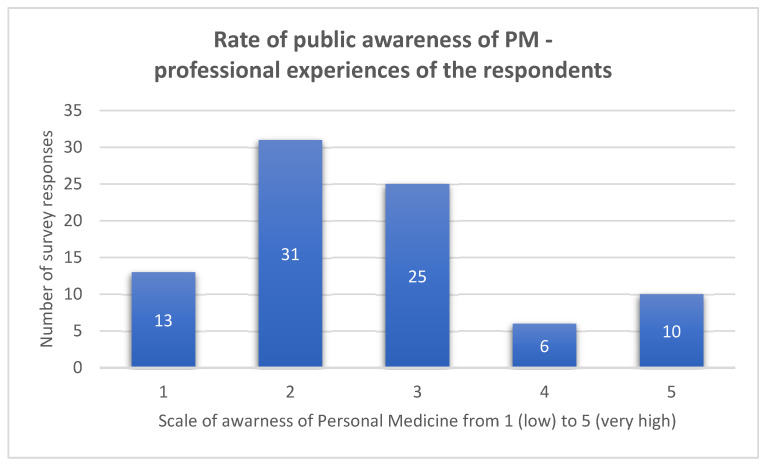
Rate of public awareness of PM—professional experiences of the respondents (survey data).

**Figure 2:**
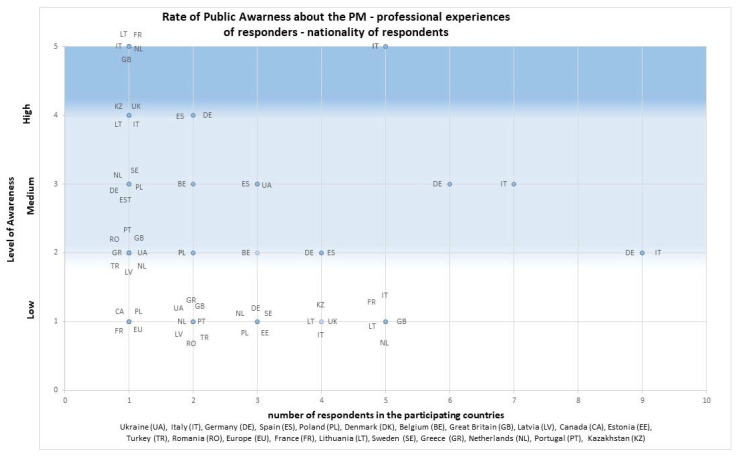
Rate of public awareness of PM—professional experiences of the respondents—breakdown by respondents’ nationality (survey data)—dot plot.

**Figure 3:**
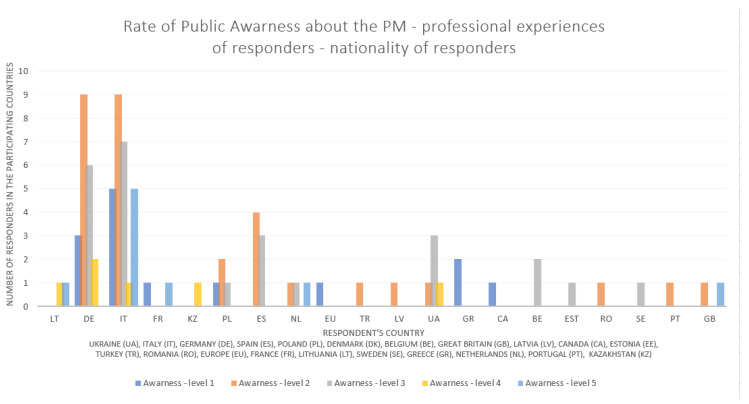
Rate of public awareness of PM—professional experiences of the respondents—breakdown by respondents’ nationality (survey data)—bar chart.

The survey shows the rate of public awareness of PM (exact question—*Given your professional experiences, please rate your public awareness of PM on a scale from 1 (low) to 5 (very high). How well are the citizens informed about PM*) Figure 1, Figure 2 and Figure 3 shows the data by the rate of public awareness of PM overall and by the nationality of the respondent.

Below, in the Table 1, the authors have presented the comparison of the public awareness of PM in a particular country of respondents, data about low, middle and high-income, and their country’s GDP per capita.

The survey also shows how difficult it is to adapt PM to the needs of citizens (exact question—*Do you think Personalised Medicine can be easily adapted to the needs of citizens?*) According to 31 respondents, adapting PM to the needs of citizens is easy, while 54 respondents pointed out the difficulty of adapting PM to the needs of citizens.

Respondents were also asked for their own ideas about what would be helpful in adapting PM to the needs of citizens—the question referred to the previous question and was—*What could be helpful? Specify* (answers in alphabetical order).

#### 4.1.1. Cooperation/Collaboration

What is crucial is close collaboration with general practitioners, more information for the citizen and support from general practitioners, cooperation between doctors working in hospitals and general practitioners. General practitioners are the first ones who can convince patients to try PM [DE_84]. Cooperation between patient advocates (they need PM), researchers and clinicians [DK_14], between industry, governments, international organisations and patient advocates [LV_23], as well as better cooperation between scientists and physicians [DE_34], is also important. There also needs to be a direct link between GPs and research hospitals [IT_69].

Collaboration between regulatory authorities and healthcare managers is also important to mutually improve awareness about the topic [IT_41] and determine common intervention plans [IT_54].

#### 4.1.2. Data Protection/IT/Data Sharing

In order to adapt PM to citizen needs, better and openly verifiable protection of PM data is important, so that patients feel confident enough to share it for their own needs and as part of aggregated data sets used for developing PM approaches for others [GB_80].

Barriers to accessing patient/citizen data should also be removed [IT_79]. Therefore, it is necessary to build data-sharing infrastructure [IT_28], because the support of technology in clinical decisions can put enormous amounts of data in the hands of physicians, facilitating their decision-making and allowing them to make the best choice for that particular patient [ES_10].

Pharmaceutical companies should commit to sharing data from all trials and clinical studies [GB_75] to increase the wide availability of data [DE_70].

#### 4.1.3. Dissemination

Respondents emphasise the importance of better disseminating knowledge about PM [IT_64], [IT_29], [UA_85], [PL_48], sharing information in the media within the Medical Society [UA_31], increasing the quality of advertisements on TV and social media [IT_11] and organising meetings with all stakeholders, including patients [IT_53].

Public awareness plans and schedules for their integration need to be developed [DE_73], [IT_74]. It is also important to disseminate scientific research findings more widely among patients and citizens [ES_40].

#### 4.1.4. Education

It is crucial to educate and discuss the concept of personal health—for a large forum of politicians and also for citizens [DE_2]. Education and cooperation between industry, governments, institutions, organisations and patient advocates [LV_23] are important. 

It is also very important to raise awareness of the benefits of PM and the education of citizens [MD_IT]. 

Therefore, it is necessary to invest in patient literacy, involvement, engagement, empowerment, introduce changes in the university education and the lifelong learning programme for professionals (doctors, nurses, others) [IT_19].

More training needs to be organised and awareness must be raised among healthcare professionals, GPs and citizens [ES_76] [IT_69], among healthcare providers and payers [DE_70]. Better education of patients on the benefits of PM is of great significance [DE_37].

#### 4.1.5. Finances

PM requires massive investments [IT_3]. Hard decisions may have to be made about the affordability of hyper-personalised medicine and the cost implications for other services. Better, more regularly updated guidelines, and evidence-based, data-driven decision support algorithms are needed to make this possible [GB_80].

The industry must improve the cost-benefit ratio;sickness funds/health insurances have to be regulated in such a way that they are interested in long-term health and cost reduction (and not just in short-term savings) and healthcare systems need to be oriented towards value-based healthcare [DE_83].

There should also be more investment in the digitisation of health data and interoperability [IT_8]. It is also important to change governance with appropriate financial incentives [EU_18].

Sustainable lighthouse projects and funding/frameworks for sustaining successful projects (these are often discontinued after the initial funding period) should be developed [DE_16].

The economics of healthcare are based on insurance and on the concept of “one-size-fits-all”. Changing the economics through PM will have an impact on the whole health system [RO_42].

In addition, telemedicine/PM services should be reimbursed by the public health system, and there should be special funds for investment in PM solutions [PL_7]. 

#### 4.1.6. Public/Citizens

Citizens might be the trigger for change. It needs to be explained to them what they would gain from PM [IT_49], and they should be engaged/involved in the decision-making process [ES_63]. It should also be known what problems healthcare providers and patients face at a local level to find the correct solutions [ES_40]. The citizen needs examples. It is necessary to create space for patients to ask, or even demand to move on to a better paradigm [IT_57].

Most importantly—patience is required, combined with a continuous effort to bring together stakeholders from many areas [DE_2] and easy-to-handle adoption strategies that help to bring new approaches into practise in an evolutionary, step-by-step process [DE_17].

#### 4.1.7. System Changes/Governmental Level

For better dissemination of knowledge about PM, a system change is very important. It will be needed if PM is implemented on a large scale, which will require a lot of effort on many levels. The biggest mistake is to expect far-reaching, short-term successes [DE_2].

National and international guidelines for implementation and reimbursement [DE_20] should be developed. Political support for a PM oriented healthcare system should be established [SB_DE], and meaningful integration of PM requires several paradigm changes, both in the public option and in decision-making [DE_45].

According to respondents, there should also be a reform of the healthcare system [NB_UA], changing the systemic approach and dialogue with public actors and HTA agencies [PL_9], and governance should be changed through appropriate financial incentives [EU_18].

The influence of vested interests on the prioritisation of certain medicines must also be overcome [GB_80].

The survey also included a question about personal opinion on existing barriers and facilitators of the implementation of the PM (exact question—*What are, in your opinion, the most important facilitators of and barriers to the public use of Personalised Medicine? What are the barriers/facilitators related (types of identified barriers to, e.g., health care system, government, PM users)? Please list and explain them briefly below*) and a question about key stakeholders, to which the previously listed information can be assigned (exact question—*In your opinion, who are the key stakeholders of the implementation barriers you have listed above.)*

In Table 2, the authors have presented the analysis and comparison of the answers they received to these specific questions.

One of the questions included in the survey concerned the need for an increasing number of trainings/conferences to introduce and present the possibility of PM (exact question—*Do you think more training/conferences should be held to introduce and show the possibility of personalised medicine? If yes, please specify the exact field*). The question was answered in the affirmative by 58 respondents, with 16 “no” responses and 5 undecided ones. 

Negative responses included explanations, such as the need for greater involvement of the mass media participation because they are more effective in shaping citizens’ opinions, while training and conferences tend to attract those who are already convinced and do not reach the general public [IT_49]. Responders think that the number of conferences should not be increased, but better ones, with a clear focus on the right audience, should be organised [ES_50], or whitepapers should be created for politicians [NL_68].

The undecided respondents also highlighted the need to raise awareness about PM, but they stressed that most people will agree that PM is good, but will not follow-up after the conference [DE_16], that it is difficult to reach the citizens [DE_62], that conferences and workshops are not the priority [DE_83], and that it is not the number of trainings/conferences that is the problem. It could well be stakeholders’ priorities and possibilities of attending the conference/workshop [SE_46].

Responders who saw the need for increasing the number of trainings/conferences to introduce and present the option of PM believe that it is important to develop such fields as:

1. Medical data-sharing practices and medical data protection;
2. Personalised exercise prescription;
3. Telemedicine;
4. Bioinformatics, artificial intelligence, genomics, machine learning, data analysis;
5. Professionals–patients relations, professionals–health managers relations, interdisciplinary and interprofessional approaches to health, emerging specialisations needed in personalised medicine (bioengineers, bionanotechnology specialists, physics applied to health, biodata analysts);
6. Health technology assessment in PM, including the patient’s perspective;
7. Oncology, internal medicine, public health, healthcare;
8. General dissemination;
9. Value-based care;
10. Paediatrics;
11. Omics and advanced diagnostic tests;
12. Health and sport;
13. Focus group working on how to transform evidence-based medicine into PM, following rational principles;
14. Benefits for the individual and the system from the thorough application of PM;
15. PM in different disease areas/specialisations, e.g., gastroenterology.

They also emphasise the need for government dialogue, which would certainly be of great value [DE_2]. They highlight the need to engage in discussion all types of stakeholders [DK_14], involving both health organisations and university experts [EU_18].

According to the respondents, it is also important to point out the successful implementation models [DE_39], provide training, and transfer the information to general practitioners, at the right time. General practitioners should be convinced. Conference concepts should enable the exchange of information between general practitioners and specialists [DE_84].

It is also important to explain the potential risks and safety of PM to citizens [IT_74].

According to respondents, there is a noticeable need for addressing training and conferences to the general public, public citizens, patient representatives and policymakers, as well as the organisation of workshops, to facilitate dialogue between public authorities/regulators/policymakers and PM clinicians/researchers [IT_41] [ES_40]. Specific training for researchers and healthcare professionals should be done in their specific health field [ES_40].

The importance of making PM user- and professional-friendly, as well as motivational and communication techniques [GR_52] are also highlighted.

## 5. Discussion

PM, as noted by Horgan D. et al., is an innovative way to produce better patient outcomes, by using an individualised or stratified approach to disease and treatment which is used as a replacement of a collective treatment approach to patients. Unfortunately, despite its tangible advantages, the process of introducing PM into the member states and European healthcare systems is delayed, due to the existing barriers to the adoption of this type of treatment [9].

### 5.1. Government and Government Agencies

European healthcare systems have inconsistent legislation and, unfortunately, working with government agencies means bureaucracy and lengthy legislative procedures [7]. PM policies and programmes also vary significantly [10].

Incorporating personalised health into existing healthcare systems is a challenge for policymakers. There is a great need to integrate personalised health into legislation. It is very important to establish regulatory frameworks to ensure cooperation and to avoid discrimination and integrate PH into existing healthcare systems. In addition, the development of regulations and standards is truly important for the regulation of the risk, defining responsibility, and protecting individuals from inequalities in personal health [11].

### 5.2. Medical Doctors/Practitioners

It is crucial to adapt the way patients and medical professionals communicate and work together to promote health. This is the only way to meet future expectations for high-quality, patient-centred care [7].

PM is mostly unknown in family medicine. It is misinterpreted as a holistic or integrative type of medicine [12].

### 5.3. Healthcare Systems

Research activity at different locations must be integrated to maximise synergies, and scientific research must be integrated with healthcare to ensure effective translation. There is also a need to harmonise scientific practices in different research sites, science and healthcare, and science, healthcare and wider society, including the ethical and regulatory frameworks, the prevailing political and cultural ethos, and patient/citizen expectations [13].

### 5.4. Healthcare Providers

Healthcare providers have a long way to the full realisation of the potential of PM. Efforts to integrate new PM technologies and practices are still in the early stages, and healthcare providers face many challenges and attribute this practice gap to the implementation of PM [14].

### 5.5. Patients

Patients need to be educated about the benefits of data-sharing [15]. Public support for the implementation of personalised medicine policies (PMPs) in routine care is important because of the high financial costs involved and the potential for diversion of resources from other services [16].

### 5.6. Industry

Pharmaceutical companies need economic incentives (high enough investment returns) to develop personalised drugs. Pricing and reimbursement policies become relevant and play a fundamental role in providing such returns on investments [4].

### 5.7. Technology Developers

The need to develop new technologies to collect and analyse data in a way that is not just linear, but integrated (understanding functioning at the system level) and dynamic (understanding the system in motion), is recognised. According to Harvey A. et al., the most important factors for developing of technologies for PM are standardisation, integration and harmonisation. The tools and all the processes for data collection and data analysis need to be standardised across research institutes [13].

### 5.8. Financial Institutions

PM can contribute to improving healthcare outcomes, as well as cost–savings, mainly by eliminating the administration of some medications to patients who are predicted to be non-responsive, although some of the personalised therapies may increase costs. Therefore, at the same time, the implementation of PM requires that health policymakers assess the potential value of these types of medicines in comparison to standard treatments for each individual indication and medical context, as the incremental health benefits of PM very often also require higher health budgets [4].

Importantly, appropriate founding and reimbursement models for PM are also very important to stimulate the development and adoption of these interventions if their clear clinical benefits can be demonstrated [17].

Also, public–private financing agreements and performance-based reimbursement models could also facilitate the development and adoption of PM interventions [17].

In addition, it is important to note that defining and measuring outcomes that demonstrate the value of PM to the parties involved, unfortunately, remains an obstacle to realising the full potential of outcomes-based reimbursement [17].

### 5.9. Media

As we can see in the paper by Hicks–Courant K. et al., in the majority of news articles PM is not clearly defined. There has also been a noticeable increase in media coverage of the benefits, rather than the risks or challenges, of PM, and reports on specific genetic tests or targeted therapies have very often appeared after their clinical utility had been established. Unfortunately, unclear information about PM in the media may contribute to patient confusion and lack of awareness [18].

Low awareness on the topic of PM in society and the lack of political support and financial investments are the main barriers. There is a clear need to broaden opportunities for critical discourse on PM, especially among policymakers. Multi-stakeholder and multi-country strategies need to be prioritised to leverage resources and expertise [10].

Some existing strategies can be implemented now (for example those which involve activities, programmes and policies, such as those related to education, awareness and patient empowerment) or can be implemented in the near future. Regrettably, some of them require stakeholders to overcome reluctance to change traditional practices and may also require a cultural change in the way medicine is approached, which is very difficult to implement [19].

As Ayers A. said, despite the challenges, PM is widely trusted to offer the best prospects for effective treatment and cure of patients with serious diseases [20].

## 6. Limitations of the Study

The analysis of barriers and facilitators presented in this paper may have some limitations. 

The number of people who participated in the survey may be considered an insufficient sample size for statistical measurement. Our research relies on sample sizes that are commonly known as small surveys, but which can provide generalizable results at the country level.

However, to avoid collecting unreliable data, questionnaires were sent to various stakeholders in the Regions4PerMed project. These included members of the Regions4PerMed project advisory board, as well as speakers at conferences and workshops (representatives from different countries and backgrounds, such as general practitioners, representatives of funding agencies, government institutions, academics, industry, etc.) and attendees at these events (the conferences were open, so there was noticeable participation from people interested in the topics). The professional profiles of the respondents are also diverse—from researchers (scientists), entrepreneurs, scientific staff, politicians, policy advisors, project managers, to doctors, civil servants, lawyers, public health experts, public health managers, biostatisticians, public health consultants, etc.

The different age range of the respondents is also striking, so that an overview of the barriers and facilitators to PM implementation from different age perspectives could be obtained.

The content of the questionnaire can also be seen as a limitation of the survey. It is important to emphasize that the content of the survey was approved by the supervisor, a professor experienced in both qualitative and quantitative research.

## 7. Conclusions

Barriers to PM adoption can be identified throughout Europe. The barriers and facilitators identified in this article need to be effectively managed in health systems across Europe. There is an urgent need to remove as many barriers as possible and create as many facilitators as possible to implement PM in the European system.

The healthcare system is currently undergoing an evolution from the traditional model, a one-size-fits-all approach, to a PM paradigm. For this reason, the evolution of healthcare toward PM requires the provision of new knowledge, a greater emphasis on the patient perspective, recognition of the value of molecular pathways in managing care, the development of new infrastructures and information management processes, and the transformation of healthcare delivery to ensure access to PM technologies and services. Addressing the challenges listed requires both short-term strategies and long-term strategies that can drive systemic and cultural change [9]. There are important interactions to consider, particularly the fact that we must first build a robust health data collection infrastructure that is widely accepted by the public, and then (later) enable data-driven evidence for effective, actionable approaches. Any relevant recommendation must clearly address this issue to make a real difference.

As Ayers (2010) said, relevant stakeholders, such as pharmaceutical and biotech companies, diagnostic companies, regulators, payers and policymakers, must work together to incentivise and remove barriers to PM. This is the only way to make this goal a reality [20]. Regrettably, European healthcare systems are only partially ready for PM adoption. If PM is to be adopted in healthcare systems, important challenges, such as Big Data integration, health literacy, reimbursement and regulatory issues still need to be addressed [21].

## Figures and Tables

**Table 1:** Comparison of the public awareness of PM in a particular country of respondents and their country’s GDP per capita.

Country	Rate of Public Awareness of PM	Number of Survey Responses	Country’s GDP (in USD) per capita (2022 Year—Estimated Data)—Source of Data—The International Monetary Fund	Division of the Countries—High-Income, Middle—Income, Low—Income Country Source of Data—World Bank List of Economies 2022
Ukraine	2	1	4862 (2021)	Lower middle income
	3	3		
	4	1		
Turkey	2	1	9961	Upper middle income
Kazakhstan	4	1	11,591	Upper middle income
Romania	2	1	15,619	Upper middle income
Poland	1	1	19,023	High income
	2	2		
	3	1		
Greece	1	2	20,876	High income
Latvia	2	1	21,482	High income
Lithuania	5	1	24,032	High income
	4	1		
Portugal	2	1	24,910	High income
Spain	2	4	29,198	High income
	3	3		
Estonia	3	1	29,344	High income
Italy	1	5	33,740	High income
	2	9		
	3	7		
	4	1		
	5	5		
European Union	1	1	37,280	
France	1	1	42,330	High income
	5	1		
United Kingdom	2	1	47,318	High income
	5	1		
Germany	1	3	48,398	High income
	2	9		
	3	6		
	4	2		
	3	2	50,598	High income
Netherlands	2	1	56,298	High income
	3	1		
	5	1		
Sweden	3	1	56,361	High income
Canada	1	1	56,794	High income

**Table 2:** Barriers and facilitators to the implementation of PM.

Key Stakeholders of the Implementation Barriers	Barriers to the Implementation of PM Interventions	Facilitators of the Implementation of PM Interventions (Ułatwienie)
Government and government agencies	- lack of clear and common public strategy on PM (IT_13) - certification and regulations (DE_24) - regional fragmentation of the health care system makes data sharing or the introduction of PM implementation policies more difficult, differences in national, regional and municipal responsibilities (SE_46), (ES_40), (IT_36), (DE_38), (ES_76), (IT_8), (IT_74) - regulatory hurdles for cross-sector innovation - collaboration (healthcare, industry, government, citizen/patient) (SE_46), (IT_41), (DE_39) - differences in national, regional and municipal responsibilities (SE_46) - delays in efficiently implementing GDPR (IT_28) - medical digital solutions are overly fragmented due to national legislations derogating from GDPR/national evaluation (FR_5) - authorities and Health Funds fear rising costs of PM today and at the same time not gaining the promised cost reductions in the future (DE_83) - traditional organisation models (IT_19) - public procurement rules (ES_50) - governance system (IT_19), (IT_60) - government in general (IT_44), (ES_40)	- government in general (IT_69) - government strategies and financing (ES_10), (ES_40), (ES_58) - government dialogue, education on the concept of personalised health for a large forum of politicians (DE_2), (IT_41) - government can contribute to making PM practice more widespread and its concept familiar to the general public (GR_27) - removing technical/legal barriers by harmonising processing of medical data (FR_5), (ES_58) - mutual recognition of digital medical solutions published in other EU member states (FR_5) - research centres and programmes, especially at the European level (IT_60) - the digitalisation of health data (ES_76) - the importance given to PM by the EC and national/regional governments (research funding, specific CSAs, national PM strategies, etc.) (ES_76) - public and private collaboration (ES_76), (ES_58)
Medical doctors/practitioners	- lack of awareness of data-intensive methods (IT_12) - lack of will of the healthcare professionals to change their current practice/accept the PM mode (IT_4), (DE_37) - medical doctors sceptical of artificial intelligence-based diagnoses (IT_12) - lack of access to individual data due to the need to guarantee their security (DE_34) - minor use of data and support by data (other than classic lab tests) (DE_37) - data used for evidence generation and clinical decision-making (quality, availability, level of impartiality) (IT_56) - lack of confidence (clarity of simulation models, repeatability, sensitivity, accuracy, etc.) (IT_56) - acceptability by the clinical world (IT_11) - lack of instructions for GP’s, lack of clear communication (IT_69) - insufficient published data (DE_32) - lack of medical doctors with training and ability to explain complex decisions regarding risk factors, efficacy and choices related to data sharing (GB_80), (LV_23) - lack of information flow from medical doctor to patient because of the work load (DE_84) - practitioners’ (MD) knowledge on new genetic testing technologies (ES_58) - lack of new specialisations in healthcare (biologists, biotechnologists, bioinformaticians) (ES_58) - lack of translation from research to clinical applications (ES_76) - lack of training of health professionals (ES_10) - lack of knowledge about the population and patients (ES_10)	- medical doctors in general (IT_44) - research hospitals (IT_69) - communicating via guidelines (DE_32) - availability of personalised data as a basis for making decision on personalised diagnosis and treatment (DE_34) - smooth and seamless cooperation between physicians and their medical knowledge and data science (DE_37) - regulations for innovation in PM and protocols in hospitals (NL_68) - dissemination of patient centered approaches, opportunities for rare disease treatment, availability of big data for real-world evidence methodologies (IT_19) - data used to generate evidence and to make clinical decisions (quality, availability, level of impartiality) (IT_56) - lack of confidence (clarity of simulation models, repeatability, sensitivity, accuracy, etc.) (IT_56)
Healthcare systems	- lack of common ontologies/dictionaries (IT_8) - lack of an integrated and digitalised information and data management system (ES_10), (GR_52), (IT_8), (PL_48), (DE_39) - lack of data availability and a guarantee of data security (DE_34), (DE_70), (IT_74), (SE_46) - lack of organisations well prepared to tackle PM (PT_18), (GR_52) - lack of PM in the daily provision of healthcare and in the public system (PL_7), (IT_13), (DE_70) - lack of incentives from the public system (PL_7) - lack of PM literacy and trained healthcare providers (ES_40), (GR_52) - translation into the healthcare system (DE_39) - lack of integration of health systems (GR_52), (SE_46) - transition to the PM system can cause dissatisfaction in patients with limited access to certain treatments considered not suitable for them (GR_27) - the fragmented medical system (DE_2) - PM needs a close interaction of Dx with PM Rx (DE_37) - lack of integration of patient related factors such as age, social background, etc. (DE_39) - system change if PM is implemented on a large scale (DE_2) - proof of concept for PM approaches (CH_DE) - disregarding prevention as a key component of PM (DE_45) - lack of investments (FR_82) - bureaucracy (IT_11) - lack of tools and approaches in medical practice (DE_2) - lack of demonstration projects and pilots (DE_70) - existing therapies are not yet personalised (DE_37) - insufficient socio-economic demonstration of value (FR_82) - aiming to contain the costs by health administrators (RO_42) - excessive focus on technology (PT_18)	- ethical issues around data privacy/ownership (DE_37) - public health care system in general (IT_28)
Healthcare providers	- lack of awareness and evidence of the benefits of PM (which is key to its large-scale adoption) (IT_49) - introducing radically new ways of dealing with health and treating diseases requires time (IT_49) - lack of awareness of PM approaches of healthcare providers (DE_70) - lack of a clear vision of what PM is (from only genomic medicine to the whole picture, including exposure/environmental/behavioural factors). (DE_16) - organisation of healthcare in general (FR_82) - dialogue between stakeholders (IT_53)	- direct contact with patients and better explanation of the benefits of a PM treatment by healthcare providers (IT_13)
Patients and patient organisations	- lack of health literacy (DE_62) - lack of knowledge about available options (DE_62) - lack of public demand for PM (DE_62) - access to PM in the public system (PL_7), (DE_51) - skills of elderly people (IT_11) - concerns about the privacy of data (DE_2) - lack of public awareness (IT_15), (DE_2), (GR_52), (LV_23) - lack of PM literacy among citizens (ES_40), (ES_25) - lack of familiarisation with technology (GR_52) - lack of user-friendly PM applications (GR_52) - PM concept difficult to understand (IT_54), (IT_57), (GB_80), (DE_16) - health literacy, general literacy (IT_15) - patients as PM users should provide their experience (DE_84) - fear of sharing a large amount of personal information with healthcare providers and the industry, GDPR (IT_4), (IT_19), (FE_5) - failure to popularise the patient’s responsibility for their own health and its management; too little incentive for the patient to strengthen their responsibility their health (PL_48) - involvement of patients in PM-related decisions and discussions is far too late and too little, often offered only as a last resort (DE_45) - low education in the field of PM (ES_25)	- patients’ associations that can represent the patients and citizens (ES_40), (IT_64) - patients’ charities and organisations, funding agencies (GB_75) - involvement (on a larger scale) of patient associations (DE_2) - education and discussion on the concept of citizens’ personal health (DE_2) - positive patients’ attitude (PL_22) - PM users can contribute to the dissemination of the concept and their personal experience (GR_27) - acceptance of personal data usage (DE_37) - proximity of patients and health care professionals and patient organisations, patient empowerment (ES_76) - willingness to use PM (TR_21) - communication and informing citizens of the benefits of PM (EE + 35)
Medical sector, scientific community, researchers, stakeholders	- lack of dialogue between stakeholders (IT_53) - concerns about the meaning for the relationship between patients and doctors, communication between doctors and patients (DE_2) - psychological hurdles for cross-sector innovation collaboration (healthcare, industry, government, citizen/patient). (SE_46) - lack of education of doctors and patients (DE_2), (BE_33), (IT_71), (IT_72), (ES_63) - low level of awareness among both practitioners and patients (UA_31), (DE_78), (IT_13) - lack of trust (DE_2) - education in general (PL_7) - lack of research, issues with recruitment of participants for PM clinical trials (BE_33), (ES_25) - lack of validation in diverse populations (GB_75) - lack of data sharing across academia (GB_75) - lack of willingness to introduce changes and to see advantages for practitioners and/or the patients (IT_57) - insufficient published data (DE_32) - lack of people with skills and knowledge (EU_18)	- the doctors and the informed patients (RO_42) - local molecular tumour conferences (DE_20) - digital platforms with data (DE_20) - need for individualised diagnostics and therapies (DE_39)
Industry	- lack of infrastructure (DE_2) - lack of standardised genomic data collection (IT_28) - lack of data sharing across industry (GB_75), (IT_28) - lack of infrastructure and homogenised regulations available to enable stakeholders to provide PM services (DE_16) - lack of a clear vision of what PM is (from only genomic medicine to the whole picture, including exposure/environmental/behavioural factors) (DE_16) - lack of therapies which are actually personalised at a sufficient scale (still a lot of blockbuster mentality and business models in the pharmaceutical industry) (DE_37) - transition to PM will require shutting down existing production infrastructure and building a new one from scratch (IT_4) - lack of PM strategies and business models at pharmaceutical companies (DE_37) - the medical industrial complex relies on revenues and profits from the existing mode of operation (DK_14) - PM needs a close interaction of Dx with PM Rx (DE_37) - lack of decision-making using the genomic data on an individual level (DE_37) - industry looking for lowest costs (DE_24) - investments in general (FR_82) - lack of will of the industry to make investments before reaching certainty of their repayment (IT_4) - lack of harmonisation between policymakers, healthcare systems, end users (IT_72)	- availability of big data for real-world evidence methodologies (IT_19) - business model consideration in conflict with classic pharmaceutical business (DE_37)
Technology developers	- lack of user-friendly technology (IT_19) - technology assessment (IT_56) - lack of algorithms and structured architecture for PM and an easy user interface (TR_21) - lack of system linking electronic patient records collected by individual doctors/medical facilities (IT system) (PL_48)	- technological centres (bridging the gap for new developments) (ES_50) - technology assessment (IT_56) - artificial intelligence technologies (IT_19) - digital platforms with data (DE_20) - technology in general (DE_38) - ICT development, lower price of technologies (IT_11)
Financial institutions	- lack of funding, lack of proper reimbursement schemes/models (LT_1), (DE_73), (IT_56), (LV_23), (KZ_6), (IT_44), (DE_20), (DE_2), (IT_4), (DE_70), (IT_47), (DE_84), (PL_22), (UA_85), (IT_19) - cost of therapies & related NHS sustainability (IT_36) - time for diagnostics/therapy validation vs. costs (IT_36) - health insurance (seeking low costs) (DE_24) - cost for health care system and society (DE_66), (ES_40) - authorities and health funds fear rising costs for PM today and not gaining the promised cost reductions in the future (DE_83) - lack of financing for start-ups, for risky and ambitious projects and, in general, for R&D (DE_24) - the economic evaluation (GR_27) - cost–benefit studies (IT_12) - evaluation/reimbursement is seen as a must for medical digital solutions (FR_5) - lack of financial incentives for HCPs to experiment with PM solutions (FR_5) - fear of discrimination at insurance and employment levels (ES_25) - economic healthcare system difficulties (financial burden, lack of insurance support (ES_25), (BE_33)	- reimbursement policy definition (IT_56) - centralised evaluation system and transparency between reimbursement prices of national health care systems (FR_5) - new purchasing methodologies (i.e., value-based reimbursement) (ES_58)
Media	- insufficient use of social media (IT_44)	- traditional media (TV and newspaper) (IT_44) - media campaign, including documentaries, to make the vision more accessible to the general public (DE_2)

## Data Availability

Restrictions apply to the availability of these data. Data was obtained from third party and are available from the authors only as anonymised version.

## Supplementary Materials

The following supporting information can be downloaded at: https://www.mdpi.com/article/10.3390/jpm13020203/s1, Supplementary Materials File S1: Questionnaire “Barriers and facilitators of Personalised Medicine implementation—qualitative study under Regions4PerMed (H2020) project”
