# Supplementary material for: Barriers and Facilitators to the Implementation of Personalised Medicine across Europe
Source: J Pers Med. 2023 Jan 23;13(2):203. doi: 10.3390/jpm13020203 (PMC9965772; doi:10.3390/jpm13020203)
Supplement: Supplementary file 1 [file jpm-13-00203-s001.zip › jpm-2148651-supplementary.pdf]

BARRIERS AND FACILITATORS OF PERSONALISED MEDICINE IMPLEMENTATION– QUALITITVE STUDY
CONCEPT UNDER REGIONS4PERMED (H2020) PROJECT

I am a PhD candidate researching the barriers and facilitators of Personalised Medicine (PM) implementation. This survey is designed to define the national and common, at the European level, barriers to and facilitators of the implementation of Personalised Medicine.

I would like to understand your experiences connected with the implementation of Personalised Medicine (PM) and learn from your knowledge. This survey aims to collect information concerning:

- your knowledge, attitudes, and practices in terms of increasing awareness and understanding PM;
- barriers and facilitators of PM implementation.

Thanks to this information, I will be able to develop strategies aimed at reducing barriers and strengthening facilitators.

By answering the following questions, you will help me understand your views and experiences. Please answer all the questions.

The following questions are aimed to gather general information.

---

1. What is your age?..........................
2. What is your gender?

   ☐male ☐female ☐other

3. What is your nationality? ……………………..
4. What is your current profession? ………………………………….
5. Are you associated with the health care system, government, or industry, or are you a Personalised Medicine user? ……………………………………..

Barriers to and facilitators of implementing Personalised Medicine

---

1. Given your professional experiences, please rate your public awareness of PM from 1 (low) to 5 (very high). How well are the citizens informed about PM?

   low … high

1	2	3	4	5

2. What are, in your opinion, the most important facilitators of and barriers to the public use of Personalised Medicine? What are the barriers/facilitators related (types of identified barriers to, e.g., health care system, government, PM users)? Please list and explain them briefly below.

Types of Barriers/Facilitators	Barriers	Facilitators

☐

The area of the implementation

1. What kind of difficulties related to PM implementation do you see?

   ……………………………………………………………………………………………………
   …………………………………………………………………………………………………....

2. In your opinion, who are the key stakeholders of the implementation barriers you have listed above?

   ……………………………………………………………………………………………………
   ……………………………………………………………………………………………………
   ……………………………………………………………………………………………………

3. Do you think that the PM can be easily adapted to citizen needs? ☐Yes ☐No

   What could be helpful? Specify.

……………………………………………………………………………………………………
…………………………………………………………………………………………….........
....

4. Do you think more training/conferences should be held to introduce and show the possibility of personalised medicine? If yes, please specify the exact field.

……………………………………………………………………………………………………
……………………………………………………………………………………………………
…………………………………………………………………………………….............

THANK YOU FOR YOUR VALUABLE INPUT
